# Editorial: The use of image and imaging flow cytometry as a tool to study host-pathogen interactions

**DOI:** 10.3389/fcimb.2022.1069960

**Published:** 2022-11-17

**Authors:** Dominic C. Jenner, Aja M. Rieger, Ziv Porat

**Affiliations:** ^1^ Defence Science Technology Laboratory, CBR Division, Porton Down, Wiltshire, United Kingdom; ^2^ Faculty of Medicine & Dentistry, University of Alberta, Edmonton, Canada; ^3^ Life Sciences Core Facilities, Weizmann Institute of Science, Rehovot, Israel

**Keywords:** imaging, cytometry, imaging cytometry, bacteria, virus, parasite

The field of cytometry is defined by the use of different technologies to study cells at the single cell level. Over recent years the tools and technologies that fall into the category of cytometry has become vast. This Research Topic was designed to identify those using imaging and imaging flow cytometry (IFC) methods to study host pathogen interactions or pathogens alone. Studying both the pathogen and/or its interaction with the host is an important part of how we increase our understanding of the effects these pathogens have on their niche environments. These studies in turn leads to a better understanding of pathogens, and their impact on their own niche, be it in the host or environmental and aids our ability to develop methods to treat (for infectious agents) or clean up (for environmental agents) these pathogens. Although there are no examples within this Research Topic it is also possible to use the same techniques to study non-pathogenic host microbe interaction such as commensal and symbiotic microbes. With the advent of multiplex imaging techniques and high parameter imaging, the upcoming years are going to be of great importance in the field of imaging. Turning multiplex and high parameter imaging into quantitative data driven science is the key to utilising these techniques, an uphill climb it might be but we are sure there are many researchers who are up for the challenge.

The articles presented in this Research Topic cover a range of different pathogens including parasites, viruses and bacteria. With research driven papers focusing on antimicrobial resistance, extracellular vesicles and bacterial phenotypes, these are great examples of how imaging is a tool at the cutting edge of pathogen research. Included also are two review papers on a timely topic of viruses, again a very important topic and one in which imaging will play a vital role in the future of pathogen-based research.

In their paper “*The Application of Imaging Flow Cytometry for Characterisation and Quantification of Bacterial Phenotypes*”, Power et al. used imaging flow cytometry to quantify growth phenotypes of various bacteria and developed a mechanism by which they could discriminate between lysed cells, damaged cells, persister cells, and viable but not culturable cells. In their work, they highlight how using IFC to monitor, quantify, and identify discrete bacterial phenotypes within a mixed population can provide insight into how bacteria adapt and respond to stressors. Importantly, IFC allows for discrimination of phenotypes not possible by traditional techniques, allowing for a better understanding of how bacterial morphology influences the potential for immune recognition and evasion.

Alfandari et al. utilized IFC to examine the distribution dynamics of extracellular vesicle (EVs) RNA cargo within recipient monocytes and macrophages. EVs are produced across almost all the living kingdoms and play a crucial role in cell-cell communication processes. EVs are especially important for pathogens, such as *Plasmodium falciparum* parasite, the species most likely to cause human malaria. Malaria parasites are able to modulate the host immune response from a distance *via* delivering diverse cargo components inside the EVs, such as proteins and nucleic acids. The authors built upon previous work by staining the RNA cargo of the vesicles and monitoring the signal they quantify the kinetics of its delivery and measure different parameters of the cargo’s distribution post internalization, within two different recipient cells of the immune system, monocytes vs macrophages. They found that while the level of the EV uptake is similar, the pattern of the signal for RNA cargo distribution is significantly different between these two recipient immune cells. This demonstrates that this method can be applied to study the distribution dynamics of the vesicle cargo post uptake to different types of cells and extend our understanding of the fate of cargo components post vesicle internalization in the complex interface between pathogen-derived vesicles and their host recipient cells.

Maan et al. utilized IFC to investigate the *Bacillus subtilis* colonization of *Arabidopsis thaliana* roots. *Bacillus subtilis* is a Gram-positive bacterium that protects the plant from various pathogens due to its capacity to produce an extensive repertoire of antibiotics, important for the elimination of pathogens and establishing beneficial host-associated communities. They used *B. subtilis* as a model to investigate the role of plant colonization in antibiotic production. Flow cytometry and IFC analysis supported the notion that *A. thaliana* specifically induced the transcription of the biosynthetic clusters for the non-ribosomal peptides surfactin, bacilysin, plipastatin, and the polyketide bacillaene. IFC was more robust in quantifying the inducing effects of *A. thaliana*, considering the overall heterogeneity of the population. This highlights IFC as a useful tool to study the effect of association with a plant host on bacterial gene expression. Furthermore, the common regulation of multiple biosynthetic clusters for antibiotic production by the plant can be translated to improve the performance and competitiveness of beneficial members of the plant microbiome.

The reviews from McCelland et al. (“*Imaging Flow Cytometry and Confocal Immunofluorescence Microscopy of Virus-Host Cell Interactions*”), and Deroubaix and Kramvis (“*Imaging Techniques: Essential Tools for the Study of SARS-CoV-2 Infection*”) highlight the importance of imaging techniques in the study of virology. As noted in these papers, imaging is a critical technique for studying viral entry and replication. These papers give a comprehensive overview of various imaging techniques and how they can be applied to the study the interaction of viruses with cells, as well as provide important caveats and challenges with these techniques.

The articles presented in this collection show how imaging and IFC are key tools in the biological researcher’s arsenal for studying pathogens and host pathogen interactions. As this once-niche technology becomes more widespread and approachable, and as imaging technologies themselves advance, we foresee an exciting future for imaging cytometry in the field of host pathogen interactions.

Content includes material subject to ^©^ Crown copyright (2022), Dstl. This material is licensed under the terms of the Open Government Licence except where otherwise stated. To view this licence, visit http://www.nationalarchives.gov.uk/doc/open-government-licence/version/3 or write to the Information Policy Team, The National Archives, Kew, London TW9 4DU, or email: psi@nationalarchives.gov.uk.

## Author contributions

All authors listed have made a substantial, direct, and intellectual contribution to the work and approved it for publication.

## Acknowledgments

The editors would like to thanks all the authors of each manuscript submitted to this Research Topic.

## Conflict of interest

The authors declare that the research was conducted in the absence of any commercial or financial relationships that could be construed as a potential conflict of interest.

## Publisher’s note

All claims expressed in this article are solely those of the authors and do not necessarily represent those of their affiliated organizations, or those of the publisher, the editors and the reviewers. Any product that may be evaluated in this article, or claim that may be made by its manufacturer, is not guaranteed or endorsed by the publisher.

